# Preclinical efficacy of oncolytic VSV-IFNβ in treating cancer: A systematic review

**DOI:** 10.3389/fimmu.2023.1085940

**Published:** 2023-03-31

**Authors:** Abdulaziz Molham Moglan, Omar A. Albaradie, Fares Fayez Alsayegh, Hussam Mohsen Alharbi, Yahya Marwan Samman, Mohammed M. Jalal, Nizar H. Saeedi, Ahmad Bakur Mahmoud, Almohanad A. Alkayyal

**Affiliations:** ^1^ College of Medicine, King Saud bin Abdulaziz University for Health Sciences, Jeddah, Saudi Arabia; ^2^ King Abdullah International Medical Research Center, Jeddah, Saudi Arabia; ^3^ Department of Medical Laboratory Technology, Faculty of Applied Medical Sciences, University of Tabuk, Tabuk, Saudi Arabia; ^4^ Department of Medical Laboratory Technology, College of Applied Medical Sciences, Taibah University, Almadinah Almunwarah, Saudi Arabia; ^5^ Strategic Research and Innovation Laboratories, Taibah University, Almadinah Almunwarah, Saudi Arabia; ^6^ King Abdullah International Medical Research Center, Riyadh, Saudi Arabia

**Keywords:** VSV-IFNβ, oncolytic virotherapy, systematic review, preclinical (*in vivo*) studies, VSV (vesicular stomatitis virus)

## Abstract

**Background:**

Cancer incidence and mortality are increasing rapidly worldwide, necessitating further investigation into developing and optimizing emergent cancer therapies. Oncolytic viruses such as vesicular stomatitis virus encoding interferon β (VSV-IFNβ) have attracted considerable attention, as they offer great efficacy and safety profiles. This systematic review aimed to determine and compare the efficacy profile between VSV-IFNβ and non-treatment controls in preclinical cancer models.

**Methodology:**

The Embase and Medline databases were systematically searched for relevant studies using related key terms and Medical Subject Headings (MeSH). Titles, abstracts, and full texts were screened, and data from eligible articles were extracted by two groups independently and in duplicate (two reviewers per group). Disagreements were resolved by a fifth independent reviewer. The included articles were all preclinical (translational) *in vivo* English studies that investigated and compared the efficacy profile between VSV-IFNβ and non-treatment controls in animal models. The risk of bias among the studies was assessed by two reviewers independently and in duplicate using SYRCLE’s risk-of-bias tool for animal studies; disparities were addressed by a third independent reviewer.

**Results:**

After employing relevant MeSH and key terms, we identified 1598 articles. A total of 87 articles were either duplicates or conference proceedings and were thus excluded. Following title and abstract screening, 37 articles were included in the full-text assessment. Finally, 14 studies met the eligibility criteria. Forty-two experiments from the included studies examined the potential efficacy of VSV-IFNβ through different routes of administration, including intratumoral, intraperitoneal, and intravenous routes. Thirty-seven experiments reported positive outcomes. Meanwhile, five experiments reported negative outcomes, three and two of which examined intratumoral and intravenous VSV-IFNβ administration, respectively.

**Conclusion:**

Although the majority of the included studies support the promising potential of VSV-IFNβ as an oncolytic virus, further research is necessary to ensure a safe and efficacious profile to translate its application into clinical trials.

**Systematic review registration:**

https://www.crd.york.ac.uk/PROSPERO/, identifier CRD42022335418.

## 1 Introduction

Cancer remains the leading cause of death globally. In 2020, approximately 19.3 and 10 million cancer cases and deaths were reported, respectively (1). The incidence and mortality rates of cancer are rapidly increasing worldwide (2, 3). In particular, the incidence rate is estimated to increase to 47% by 2040 (1). In most cases, traditional cancer treatments, including surgery, radiation, and chemotherapy, are insufficient to provide long-lasting protection against cancer. Accordingly, there is an urgent need to develop new cancer treatments that are more effective in killing cancer cells.

Oncolytic virotherapy is an emerging cancer treatment modality. Oncolytic viruses selectively infect and subsequently kill tumor cells while sparing normal cells (4). Vesicular stomatitis virus (VSV) is a non-segmented, negative-sense, RNA virus. Generally, VSV is non-pathogenic to humans with no preexisting immunity (5). It possesses an 11-kilobase genome, encoding for five proteins: nucleocapsid protein, phosphoprotein, matrix protein, glycoprotein, and large polymerase protein (6). Glycoprotein allows this virus to infect most mammalian cells (5). The low-density lipoprotein receptor has been identified as the cellular receptor for VSV cell entry (7).

Moreover, the matrix protein allows wild type VSV to evade innate antiviral immunity by inhibiting the cytoplasmic transport of mRNA, leading to the attenuation of the synthesis of IFN and other pro-inflammatory proteins (8). This can be mitigated by inserting the IFN-β gene into the viral genome of VSV (9). Cancer cells, which possess a defective or inactive IFN pathway, can be effectively targeted using VSV-IFNβ. Additionally, VSV-IFNβ enhances the oncoselectivity and safety of VSV, without attenuating the oncolytic profile of the virus (8–13). Interestingly, IFN-β has been shown to possess other potential properties in addition to its antiviral properties. It can boost the anti-tumor immune response, activate natural killer cells, T-cells, and professional antigen-presenting cells such as dendritic cells, and insert anti-proliferative effects as well as hindering intratumoral angiogenesis (9, 10, 14). Several preclinical studies have been conducted to determine the efficacy of VSV encoding IFNβ (VSV-IFNβ) *in vivo* (15–18). VSV-IFNβ has also been reported to be less toxic than wild-type VSV *in vivo* (9). As such, VSV-IFNβ may be a safe and effective therapeutic option, into which further investigation is required.

To our knowledge, no systematic review has been conducted on preclinical studies focusing on VSV-IFNβ. Therefore, this review aimed to evaluate and report the efficacy of VSV-IFNβ *in vivo* in various preclinical tumor models.

## 2 Materials and methods

### 2.1 Eligibility criteria

This systematic review was performed in accordance with the Preferred Reporting Items for Systematic Reviews and Meta-Analyses (PRISMA) protocol (19). Furthermore, this systematic review was conducted in compliance with a pre-specified protocol registered in PROSPERO (CRD42022335418). All preclinical *in vivo* (translational) studies that reported the efficacy of VSV-IFNβ in animal models were eligible for inclusion. Preclinical studies were defined as studies investigating medically relevant interventions performed using non-human models. The model was limited to *in vivo* experiments. Validations of only *in vitro* or *ex vivo* experiments were therefore excluded. Non-English-language publications, articles reporting unrelated data, and papers published as conference proceedings were also excluded.

### 2.2 Search strategy

**Figure 1:**
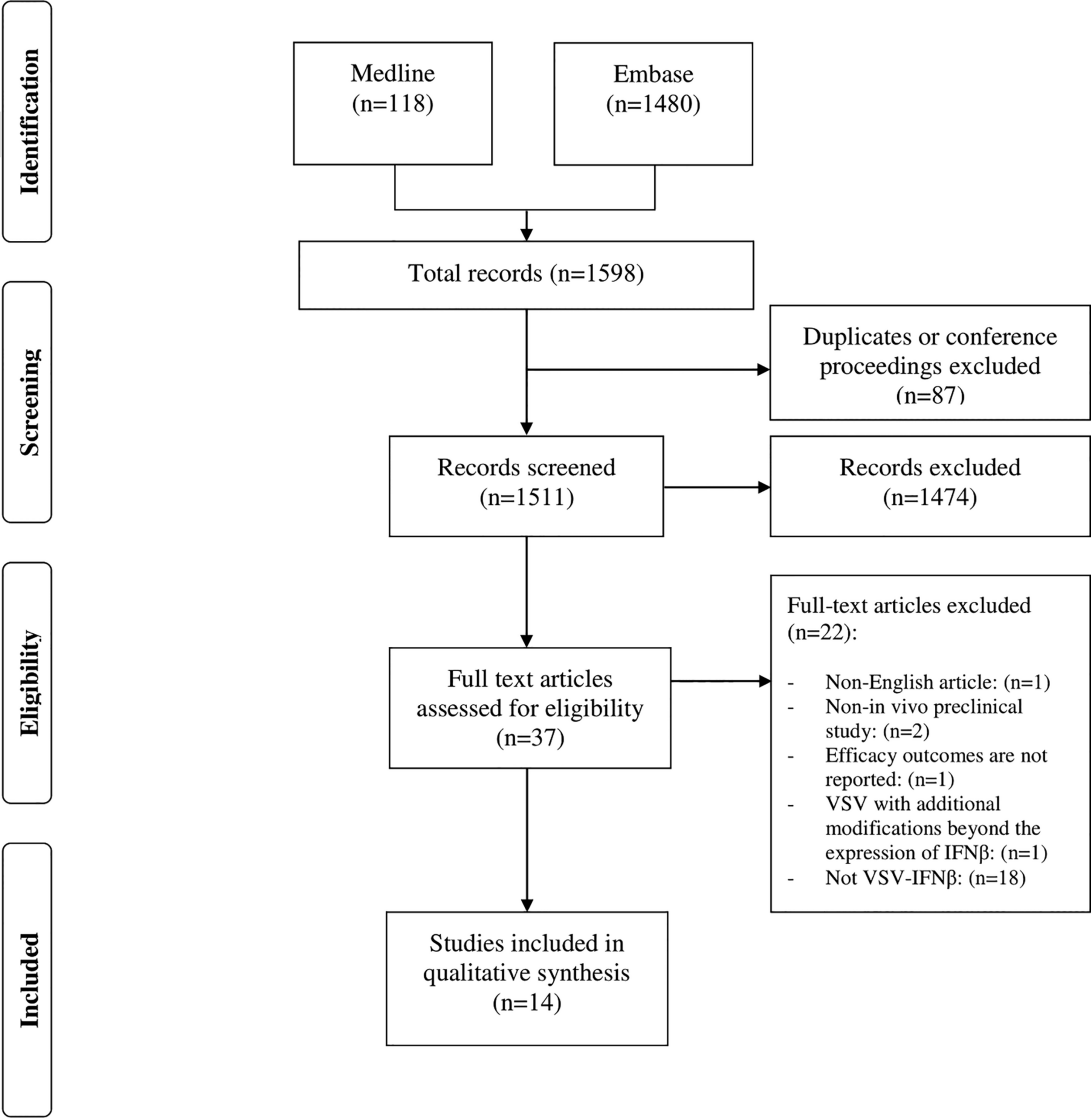
Prisma flow chart of search and selection process.

A systematic search was conducted in the Medline (PubMed) and Embase databases *via* Ovid (from 1900 to 2022 inclusively; the last search was performed in mid-March 2022). The complete systematic search and selection process is described in (**Figure 1**). The following key terms and Medical Subject Headings were used for each database when available: VSV, vesicular stomatitis Indiana virus, VSV-IFN, VSV-Interferon-Beta, VSV Interferon Beta, VSV-IFN-beta, VSV-IFN-b, VSV-IFN beta, oncolytic virotherapy, oncolytic viruses, virotherapy, virotherapy agent, cancer, malignant neoplasm, malignancy, neoplasms, breast neoplasms, breast tumor, rectal neoplasms, rectum tumor, brain neoplasms, brain tumor, lung neoplasms, lung tumor, adenocarcinoma, tumor cell line, tumor, tumor regression, tumor control, and animal models.

### 2.3 Study selection and data extraction

After title and abstract screening for eligibility, full-text assessment, and data extraction were performed independently and in duplicate by two reviewing groups (with two reviewers each); each reviewing group examined half of the search results. Disagreements were resolved by a third reviewer. The data extracted were as follows: name of the first author, year of publication, type of cancer, type of animal model, age and sex of the model, type of VSV, tumor implantation, tumor measures at virus administration, number of animal models, viral load, route of administration, number of doses, and adverse events. The desired outcomes, including tumor regression, tumor control, and/or survival rate compared with that of normal saline, were evaluated. All data were retrieved using a predefined data collection sheet.

### 2.4 Risk-of-bias assessment

The risk of bias among the included studies was assessed using Systematic Review Centre for Laboratory animal Experimentation (SYRCLE) risk-of-bias tool for animal studies (20) by two reviewers independently and in duplicate. Divergences were resolved by a third independent reviewer.

## 3 Results

### 3.1 Search results and study characteristics

A total of 1598 articles were identified from the two databases using the search criteria (Embase, n=1480; Medline, n=118). Among them, 87 were duplicates of conference proceedings and were therefore excluded. However, 37 of these referred to VSV-IFNβ in the title and abstract, the remaining 1474 articles were irrelevant, i.e., did not meet the inclusion criteria. After a full-text review, only 16 papers were selected; two of these were further excluded, resulting in a total of 14 remaining papers (**Figure 1**). One of these papers did not report efficacy outcomes, and only provided safety data. The other paper involved a modified form of VSV that has an additional modification of the virus in addition to the expression of IFN-β.

A total of 23 preclinical efficacy studies drawn from eight articles evaluated intratumoral administration of VSV-IFNβ for the treatment of established tumors. The efficacy of intratumorally administered VSV-IFNβ was assessed for the following seven types of cancer: prostate cancer (LNCaP and PC3, human; RM9, mouse), endometrial adenocarcinoma (AN3 CA, human; HEC-1-A, human), melanoma (B16F10, mouse; B16ova, murine), colon carcinoma (CT26, mouse), squamous cell carcinoma (FAT-7), non-small-cell lung cancer (LM2 urethane-induced, murine; H2009, human), and mesothelioma (AB12, murine; MSTO-211H, human; MSTO, human; REN, human). Twenty and three efficacy studies reported positive and negative outcomes, respectively.

Two studies from a single article assessed the efficacy of intraperitoneally administered VSV-IFNβ in treating pre-established mesothelioma (AB12, murine). These two studies concluded with positive outcomes.

Eight articles consisting of seventeen preclinical studies investigated the efficacy of intravenously administered VSV-IFNβ vaccines in treating implanted tumors. Tumors treated intravenously were mainly of five types, albeit with numerous cell lines: endometrial adenocarcinoma (AN3 CA, murine), lung cancer (LM2, murine; A549-Luc, human), acute myeloid leukemia (C1498, murine; C1498.GFP, murine), squamous cell carcinoma (SCC; FAT-7, rat), and plasma cell myeloma (5TGM1, murine; MPC-11, murine; KAS6/1, human). Only 2 of 17 experiments reported a negative outcome. Details of the included studies are presented in **Table 1**.

**Table 1:** Summary of the experimental details of the reviewed studies.

First Author, Year of Publication	Reference	VSV	Type of Cancer (Cell Line)	Animal Model, sex, age	Model Type, Tumor Implantation	Number of Animal Models	Viral Load, Number of Doses	Route of Administration	Outcome
Udayakumar et al., 2020	(21)	VSV-hIFNβ	Prostate cancer (PC3)	Athymic nude mice, Male, 4-8 weeks	Xenograft, SC	15	10^5^ PFU, 1	IT	–
		VSV-mIFNβ	Prostate tumor (RM9)	C57BL/6 mice, male, NM	Syngeneic, SC	13	10^5^ PFU, 1	IT	+
Liu et al., 2014	(16)	VSV-hIFNβ	Endometrial cancer (HEC-1-A)	Athymic nude mice, female, 4-5 weeks	Xenograft, SC	5 per group	10^7^ TCID50, 1	IT	+
			Endometrial cancer (AN3 CA)	Athymic nude mice, female, 4-5 weeks	Xenograft, SC	5 per group	10^7^ TCID50, 1	IT	+
			Endometrial cancer (AN3 CA)	Athymic nude mice, female, 4-5 weeks	Xenograft, SC	10 per group	10^6^ TCID50, 1	IV	+
		VSV-mIFNβ	Endometrial cancer (AN3 CA)	Athymic nude mice, female, 4-5 weeks	Xenograft, SC	10 per group	10^6^ TCID50, 1	IV	+
Patel et al., 2020	(22)	VSV-mIFNβ	lung cancer (LM2)	A/J mice, NM, 8 weeks	Syngeneic, IV	5 per group	10^8^ TCID50 or 10^6^ VSV-infected mBOECs, 6	IV	–
			lung adenocarcinoma (A549-Luc)	Fox Chase SCID beige mice, female, 8 weeks	Xenograft, IV	10 per group	10^8^ TCID50 or 10^6^ VSV-infected mBOECs, 3	IV	+
Zhang et al., 2016	(23)	VSV-mIFNβ-NIS	myeloma (KAS6/1)	CB17 ICR SCID,female, 4-5 weeks	Xenograft, SC	70	10^5^, 10^6^, 10^7^, or 10^8^ TCID50, NM	IV	+
			plasmocytoma (5TGM1)	C57BL/KaLwRij, female, 7-8	Syngeneic, SC	65	10^5^, 10^6^, 10^7^, or 10^8^ TCID50, 1	IV	+
Shen et al., 2016	(24)	VSV-mIFNβ-NIS	Acute myeloid leukemia (C1498)	C57BL/6J, female, 4-5 weeks	Syngeneic, SC	5 per group	10^6^, 10^7^, or 10^8^ TCID50, 1	IV	+
			Acute myeloid leukemia (C1498.GFP)	C57BL/6J, female, 4-5 weeks	Syngeneic, IV	11 or 12 per group	10^8^ TCID50, 1	IV	+
Willmon et al., 2009	(25)	VSV-hIFNβ	Mesothelioma (AB12)	BALB/c, SCID, NM, NM	Syngeneic, SC	8 per group	6.6×10^8^ PFU, 3	IT	–
			Mesothelioma (AB12)	BALB/c, SCID, NM, NM	Syngeneic, IP	8 per group	6.6×10^8^ PFU, 3	intraperitoneal	+
			Mesothelioma (MSTO-211H)	SCID, NM, NM	Xenograft, SC	8 per group	6.6×10^8^ PFU, 2	IT	+
		VSV-mIFNβ	Mesothelioma (AB12)	BALB/c, SCID, NM, NM	Syngeneic, SC	8 per group	6.6×10^8^ PFU, 3	IT	+
			Mesothelioma (AB12)	BALB/c, SCID, NM, NM	Syngeneic, SC	8 per group	6.6×10^8^ PFU, 2	IT	+
			Mesothelioma (AB12)	SCID, NM, NM	Syngeneic, SC	8 per group	6.6×10^8^ PFU, 2	IT	+
			Mesothelioma (MSTO-211H)	SCID, NM, NM	Xenograft, SC	8 per group	6.6×10^8^ PFU, 2	IT	+
			melanoma (B16ova)	C57Bl/6, NM, NM	Syngeneic, SC	8 per group	5×10^8^ PFU, 2	IT	+
			Mesothelioma (AB12)	BALB/c, NM, NM	Syngeneic, IP	8 per group	6.6×10^8^ PFU, 3	intraperitoneal	+
Patel et al, 2015	(15)	VSV-mIFNβ	NSCLC (H2009)	nude mice, NM, 4-6 weeks	Xenograft, SC	5 per group	5×108 TCID50, 3	IT	+
			NSCLC (LM2)	A/J mice, NM, 6 weeks	Syngeneic, SC	10 per group	1.5×10^10^ TCID50, 3	IT	+
Kurisetty et al, 2014	(17)	VSV-hIFNβ	SCC of head and neck (FAT-7)	Fischer-344 rats, female, 6-7 weeks	Syngeneic, SC	10 per group	5×10^8^ PFU, 1	IV	+
			SCC of head and neck (FAT-7)	Fischer-344 rats, female, 6-7 weeks	Syngeneic, SC	10 per group	5×10^8^ PFU, 1	IT	+
		VSV-rIFNβ	SCC of head and neck (FAT-7)	Fischer-344 rats, female, 6-7 weeks	Syngeneic, SC	10 per group	5×10^7^, 5×10^8^, 5×10^9^ PFU, 1	IT	+
			SCC of head and neck (FAT-7)	Fischer-344 rats, female, 6-7 weeks	Syngeneic, SC	10 per group	5×10^8^ PFU, 1 and 2	IT	+
			SCC of head and neck (FAT-7)	Fischer-344 rats, female, 6-7 weeks	Syngeneic, SC	10 per group	5×10^8^ PFU, 1	IV	+
Saloura et al, 2010	(26)	VSV-hIFNβ	pleural mesothelioma (REN)	SCID, NM, NM	Xenograft, SC	6-8 per group	6.6×10^8^, 3	IT	–
			pleural mesothelioma (MSTO)	SCID, NM, NM	Xenograft, SC	6-8 per group	6.6×10^8^, 3	IT	+
		VSV-mIFNβ	pleural mesothelioma (REN)	SCID, NM, NM	Xenograft, SC	6-8 per group	6.6×10^8^, 3	IT	+
			pleural mesothelioma (MSTO)	SCID, NM, NM	Xenograft, SC	6-8 per group	6.6×10^8^, 3	IT	+
Patel et al, 2019	(27)	VSV-mIFNβ	NSCLC (LM2)	A/J mice, NM, 8 weeks	Syngeneic, SC	10 per group	1.5×10^9^ TCID50, 3	IT	+
Durham et al, 2017	(28)	VSV-mIFNβ	melanoma (B16-F10)	C57BL/6 mice, female, 6-8 weeks	Syngeneic, SC	NM	10^9^ TCID50, 4	IT	+
			colorectal carcinom (CT26)	BALB/c mice, female, 6-8 weeks	Syngeneic, SC	11	10^9^ TCID50, 4	IT	+
Naik et al, 2012	(8)	VSV-hIFNβ	myelom (5TGM1)	C57Bl/KaLwRij mice, female, 4-6 weeks	Syngeneic, SC	NM	10^8^ TCID50, 1	IV	+
			myelom (5TGM1)	C57Bl/KaLwRij mice, female, 4-6 weeks	Syngeneic, IV	26 per group	5×10^7^ TCID50, 1	IV	+
		VSV-mIFNβ	myelom (5TGM1)	C57Bl/KaLwRij mice, female, 4-6 weeks	Syngeneic, SC	NM	10^8^ TCID50, 1	IV	+
			myelom (5TGM1)	C57Bl/KaLwRij mice, female, 4-6 weeks	Syngeneic, IV	26 per group	5×10^7^ TCID50, 1	IV	+
Zhang et al, 2016	(29)	VSV-mIFNβ-NIS	myeloma (MBC-11 Plasmacytoma)	BALB/c mice, female, 5-6 weeks	Syngeneic, SC	13 per group	2×10^6^, 2×10^7^, 2×10^8^ TCID50, 1	IV	+
Yarde et al, 2013	(18)	VSV-mIFNβ-NIS	Meningeal Myloma (5TGM1)	C57BL/KaLwRijHsd mice, Female, NM	Syngeneic, SC	10	10^8^ TCID50, 1	IV	+
			Meningeal Myloma (5TGM1)	C57BL/KaLwRijHsd mice, Female, NM	Syngeneic, IV	5	10^8^ TCID50, 1	IV	–

NSCLC, Non-Small Cell Lung Cancer; SCC, Squamous Cell Carcinoma; NM, Not Mentioned; IV, Intravenous; SC, Subcutaneous; IP, Intraperitoneal; IT, Intratumoral; PFU, Plaque-forming units; TCID50, Median Tissue Culture Infectious Dose; mBOECs, murine blood outgrouth endothelial cells; +, the study reported a positive outcome; -, the study reported a negative outcome.

### 3.2 Risk-of-bias assessment

Most included studies had an unclear risk of bias (**Table 2**). Reporting of randomization, blinding, and animal housing was inadequate. Only two studies (14%) reported the allocation of animals to different randomized groups to minimize selection bias (28, 29). Eleven studies (79%) reported an adequate amount of information regarding animal characteristics upon intervention, which mitigated the risk of selection bias. All studies included sufficient details regarding outcome evaluation. As most information required to assess the included studies was not available, evaluation using SYRCLE’s risk-of-bias tool was not recommended.

**Table 2:** Assessment of risk of bias using SYRCLE’S risk of bias tool.

First Author, Year of Publication	1. Was the allocation sequence adequately generated and applied?	2. Were the groups similar at baseline or adjusted for confounders?	3. Was the allocation adequately concealed?	4. Are the animals randomly housed during the experiment?	5. Were the caregivers/investigators adequately blinded during the course of the experiment?	6. Were animals selected at random during outcome assessment?	7. Was the outcome assessor adequately blinded?	8. Were incomplete outcome data adequately addressed?	9. Is the study free of selective outcome reporting?	10. Was the study apparently free of other problems that could cause a high risk of bias?
Liu et al. (16)	U	Y	U	U	U	U	U	U	Y	Y
Udayakumar et al. (21)	U	Y	U	U	U	U	U	U	Y	Y
Patel et al. (22)	U	N	U	U	U	U	U	U	Y	Y
Zhang et al. (29)	U	Y	U	U	U	U	U	U	Y	Y
Shen et al. (24)	U	Y	U	U	U	U	U	U	Y	Y
Willmon et al. (25)	U	Y	U	U	U	U	U	U	Y	Y
Patel et al. (15)	U	Y	U	U	U	U	U	U	Y	Y
Kurisetty et al. (17)	U	Y	U	U	U	U	U	U	Y	Y
Saloura et al. (26)	U	N	U	U	U	U	U	U	Y	Y
Patel et al. (27)	U	Y	U	U	U	U	U	U	Y	Y
Durham et al. (28)	Y	Y	U	U	U	U	U	U	Y	Y
Naik et al. (8)	U	Y	U	U	U	U	U	U	Y	Y
Zhang et al. (29)	Y	Y	U	U	U	U	U	U	Y	Y
Yarde et al., (18)	U	N	U	U	U	U	U	U	Y	Y

Responses to signaling questions of each domain were judged as follows: Y; (Yes) low risk of bias, N; (No) high risk of bias, U; Unclear risk of bias.

### 3.3 Intratumorally administered treatment

#### 3.3.1 Prostate cancer

Athymic nude mice were subcutaneously implanted with LNCaP and PC3 prostate cancer cell lines. Both LNCaP and PC3 xenografts were injected with VSV-hIFNβ but at different plaque-forming units (PFUs) (LNCaP at 1×10^1^ PFU and PC3 at 1×10^5^ PFU). P-values were not reported; instead, the prostate-specific antigen (PSA) level and tumor volume (TV) were provided as a surrogate for efficacy. The VSV-hIFNβ-administered LNCaP models showed a PSA level of 88.2 ± 16.68 ng/mL compared with the heat-inactivated viral control (PSA level, 75.96 ± 14.78 ng/mL). Furthermore, the VSV-hIFNβ-administered PC3 models had a TV of 873.32 ± 132.43 mm^3^ in comparison with the heat-inactivated virus or untreated group (622.76 ± 136.62 or 671.69 ± 125.99 mm^3^, respectively) (21). Even though the low dose of VSV-hIFNβ 1×10^1^ PFU did not improve the treatment outcomes as a single agent, the higher dose at 1×10^5^ PFU resulted in an improved survival benefit as evident by the PSA levels suggesting that the treatment of VSV-hIFNβ alone but at high dose is required to exert the hIFNβ effect on the tumor cells. Further investigation on the tumor microenvironment in the presence of hIFNβ expression would be needed to fully understand the mechanisms behind the observed differences in treatment outcomes.

Another prostate cancer model was investigated by implanting a RM9 prostate cancer cell line subcutaneously in C57BL/6 mice, which were treated with VSV-mIFNβ at 1×10^5^ PFU. The TVs were as follows: 541.36 ± 36.62 mm^3^ for VSV-mIFNβ and 832.44 ± 135.29 mm^3^ for no-treatment (21). Even though the treatment with VSV-mIFNβ significantly attenuated the tumor progression compared to the no-treatment group, there was no complete tumor regression reported in the study. This is supported by the fact that there was no statistically significant increase in the number of CD8+ T cells following the VSV-mIFNβ treatment.

#### 3.3.2 Endometrial adenocarcinoma

The cell lines (AN3 CA and HEC-1-A) were subcutaneously established in athymic mice. Either a single dose of VSV-hIFNβ at 1×10^7^ median tissue culture infective dose (TCID_50_) or saline control was administered (five mice per group in each tumor model). The mice in the saline-injected group were euthanized owing to ulceration or exceeding tumor burden, while 5 out of 10 mice in both tumor models were euthanized owing to neurotoxic signs (weight loss or paralysis). Regardless of this neurotoxic complication, the experiments indicated that three mice (two induced with HEC-1-A and one with AN3 CA) had complete tumor regression. Despite successfully controlling the tumors, the survival rate was similar in both; the group treated with VSV-hIFNβ and the group given only saline, due to the early euthanasia (16).

#### 3.3.3 Melanoma

B16F10 tumors were subcutaneously implanted in C57BL/6 mice. After tumors reached a volume of 200 mm^3^, two doses of VSV-mIFNβ per week (a total of four doses) were administered at 1×10^9^ TCID_50_. Tumor growth was delayed; however, no tumor regression was noted. The median overall survival (mOS) of 23 days was prolonged compared with that in the sham control and heat-inactivated VSV-mIFNβ (28).

Another study experimented with the B16-OVA tumor cell line, which was implanted into C57Bl/6 mice. When the tumors reached a volume of around 200 mm^3^, a weekly dose of VSV-mIFNβ at 5×10^8^ PFU or saline was injected in each group (n=8) for 2 weeks. The administration of VSV-mIFNβ led to significant tumor regressions (p<0.05) in comparison with saline control (25).

The B16F10-OVA tumor model may be more responsive to treatment with VSV-mIFNβ and potentially more immunogenic due to the presence of the OVA protein (30, 31). OVA is a known immunogen and the presence of this protein in the B16F10-OVA cells may stimulate a stronger immune response compared to the unmodified B16F10 cells (30, 31). This heightened immune response may be more effective at attacking and eliminating cancer cells, leading to greater tumor regressions when treated with VSV-mIFNβ (30, 31). Additionally, the OVA protein acts as a target for the immune system, allowing it to more effectively recognize and attack the cancer cells (30, 31). This makes the B16F10-OVA cells more sensitive to the immune-mediated effects of VSV-mIFNβ (30, 31). However, it is important to note that the immunogenicity and response to treatment can be influenced by other factors such as the time of treatment and the tumor size at the treatment initiation which influence the immunosuppressive tumor microenvironment. As such, further research would be needed to confirm these potential mechanisms (30, 31).

#### 3.3.4 Colon carcinoma

CT26 colon cancer was established subcutaneously in BALB/c mice. After the volume of the tumor reached 200 mm^3^, two doses per week for a total of four doses of VSV-mIFNβ at 1×10^9^ TCID_50_ were administered intratumorally. The control groups were sham and heat-inactivated VSV-mIFNβ. Tumor regression was present in 2 out of 11 mice treated with VSV-mIFNβ, while a small increase in the mOS was observed in the VSV-mIFNβ treatment group compared with that in the sham (p<0.05) and heat-inactivated VSV-mIFNβ control groups (mOS=25, 18, and 19.5 days, respectively) (28). In this study, VSV-mIFNβ was not effective in producing antigen-specific immune responses; specifically the AH-1-specific responses in the CT26 model. However, when combined with either αPD-L1 or αCTLA-4, it significantly increased AH-1 responses. It is suggested that the resistance to VSV-mIFNβ therapy in this model may be caused by Tregs-induced immunosuppression.

#### 3.3.5 Non-small-cell lung cancer

LM2 urethane-induced tumors were established in 8-week-old A/J mice, and treatment was started 10 days after tumor injection (approximately 0.5 cm^3^) by administering three doses of 1.5×10^9^ TCID_50_ of VSV-mIFNβ with a volume of 0.1 mL on alternate days. The experimental groups were PBS+DMSO, PBS+ruxolitinib, VSV-mIFNβ+DMSO, and VSV-mIFNβ+ruxolitinib, a JAK/STAT pathway inhibitor, which has been reported to exert a reverse effect on IFN signaling, therefore enhancing the oncolytic effects of the virotherapy (30). A significant (p<0.05) difference was observed in the TV between the animals treated with PBS and DMSO and those treated with VSV-IFNβ alone or in combination with ruxolitinib (27).

Ten A/J mice bearing urethane-induced LM2 tumors were treated with PBS or VSV-mIFNβ at 1.5×10^10^ TCID_50_ every other day for a total of three doses. The anti-tumor effects of VSV-mIFNβ were seen with a clear distinction in the TV curve by day 5 (p<0.001), with 30% (out of n=10) showing complete tumor regression. Moreover, the overall survival of the VSV-mIFNβ-treated mice was significant (p<0.001) (15).

An additional model was tested for the efficacy of VSV-mIFNβ on non-small-cell lung cancer. Nude mice bearing H2009 tumors were injected at a dose of 1×10^8^ PBS or VSV-mIFNβ once a week for 3 weeks (a total of three doses) at 5×10^8^ or 6.6×10^8^ TCID_50_. The administration of two different doses led to varying results, with VSV-mIFNβ at 6.6×10^8^ TCID_50_ showing anti-tumor activity in the H2009 model, although these results did not achieve statistical significance. However, VSV-mIFNβ treatment at 5×10^8^ TCID_50_ showed that the TV curves separated early and significantly differed between the treated and untreated mice from day 7 onwards (p<0.01 for each time point after treatment) (15).

#### 3.3.6 Squamous cell carcinoma

Fischer 344 rats with subcutaneously implanted FAT-7 SCC were utilized for testing the anti-tumor effects of VSV-rIFNβ. *In vivo* treatment was initiated after the tumor diameter was measured (0.5 cm), and the decision to euthanize was made following evidence of neurotoxicity, TV exceeding 10% of body weight, tumor ulceration, inability to consume food and water, or 15% of body weight loss. Three different doses of VSV-rIFNβ (5×10^7^, 5×10^8^, and 5×10^9^ PFUs) were injected intratumorally. At 5×10^8^ PFU, the treated group had the most significant tumor growth delay and time to euthanization (p<0.0001 on day 43 and p=0.0008, respectively) compared with the control group (17).

The higher dose did not yield any additional effects on the reduction in tumor growth delay or time to euthanization. Single and dual doses of VSV-rIFNβ were compared at 5×10^8^ PFU. Both doses significantly delayed tumor growth (p<0.0001 on day 43) and extended the time to euthanization (p<0.05) compared with controls (17).

Moreover, the effects of the injection of VSV-hIFNβ on the syngeneic model of SCC were investigated. A single-dose treatment with VSV-hIFNβ at 5×10^8^ PFU was initiated around 21 days after tumor establishment, and the tumor growth-delaying effects were significant (p=0.0002 VSV-intratumoral vs. control; p=0.0004) in addition to the overall improvement in the time to euthanization in comparison with those of the mock-treated group (p<0.0001) (17).

#### 3.3.7 Mesothelioma

AB12 tumors were implanted into the flank of BALB/c mice; when the tumors reached a diameter of 200 mm^3^, a weekly dose of PBS or 6.6×10^8^ PFU of human and murine IFNβ-integrated VSV was administered for 3 weeks. Euthanization was performed when the tumor burden exceeded 1500 mm^3^ or when signs of toxicity were evident. VSV-mIFNβ injection into AB12 tumors significantly improved the antitumor effects compared with the control (p<0.01); however, no significant effect of VSV-hIFNβ therapy over PBS control (p=0.27) was evident. Investigations into the BALB/c model showed that the survival rate of the VSV-mIFNβ-treated mice significantly increased, with four out of eight mice cured of tumors compared with a tumor progression in eight out of eight mice in the PBS-treated group. However, all these long-term survivors failed a subsequent rechallenge of AB12 cells (25).

SCID and SCID CD8+T cell-depleted models were also investigated to evaluate the efficacy of both VSV-hIFNβ and VSV-mIFNβ. After AB12 and MSTO-211H tumors were implanted in the flanks of both models and reached a volume of 200 mm^3^, two doses of VSV-mIFNβ or VSV-hIFNβ at 6.6×10^8^ PFU were administered in a one-dose-per-week treatment regimen. VSV-mIFNβ treatment in SCID mice reduced the growth rate of AB12 significantly (p<0.001) compared with the control. AB12 tumors developed more rapidly in the SCID mice than in the BALB/c mice. Therefore, tumor growth inhibition significantly differed between the BALB/c and SCID mice (p<0.0001); the BALB/c mice exhibited 60% growth inhibition, while the SCID mice exhibited approximately 35% tumor growth inhibition. Meanwhile, the mice depleted of CD8+T cells and treated with VSV-IFNh grew tumors approximately 25% more slowly than did the mice treated with controls. Similarly, the treated BALB/c mice exhibited 60% inhibition of tumor growth (p<0.0001). These data indicate that approximately half of the intratumoral infusion of VSV-IFNh is dependent on an intact CD8+T cell compartment (25).

For MTSO tumor-bearing SCID mice, PBS, VSV-hIFNβ, or VSV-mIFNβ was injected at a dose of 6.6×10^8^ PFU. All VSV-hIFNβ-treated groups showed significant evidence of tumor regression compared with the untreated group; however, these mice exhibited intolerable neurotoxicity 40 days after tumor implantation. In contrast, the VSV-mIFNβ-treated tumors up to 40 days after tumor seeding showed similar levels of tumor growth inhibition, but which was not associated with viral toxicity. Administration of VSV-hIFNβ or VSV-mIFNβ significantly inhibited tumor growth in the treatment group compared with the control (p<0.0001 for both) in MTSO tumors (25).

Saloura et al. examined the efficacy of VSV-IFNβ in xenograft models of mesothelioma (31). Three groups of SCID mice (n=6-8 per group) were implanted with REN or MSTO mesothelioma cells in the hind flank. Once the tumors reached approximately 200 mm3 in size, the mice were treated with a control medium, VSV-hIFNβ, or VSV-mIFNβ at 6.6 x 108 PFU, given intratumorally once a week for 3 consecutive weeks. VSV-mIFN-β served as a control for VSV-hIFN-β because the IFN proteins do not have any cross reactivity between species. Interferon-beta has been shown to have both direct antiproliferative effects on tumor cells and the ability to stimulate the immune system, including activating natural killer (NK) cells and inducing the production of proinflammatory cytokines. The MSTO xenografts treated with either VSV-hIFNβ or VSV-mIFNβ showed significant (p<0.05) tumor growth inhibition (approximately 75%) compared to the controls. However, in the hIFNβ resistant REN model, the group treated with VSV-hIFNβ showed insignificant tumor regression compared to the control group (p=0.2). In contrast, the group treated with VSV-mIFNβ showed significant tumor regression of approximately 80% compared to the mock treatment group (p<0.01) (26). The results of studies that used intratumoral treatment of VSV-IFNβ are summarized in **Table 3**.

**Table 3:** Summary of the studies that utilized intratumoral treatment.

Tumour type (cell line)	Animal model (X/S)	Number of seeded tumor cells (route)	Time of treatment in relation to tumor implantation	Treatment conncetratoin	Number of doses	Survival benefits (P-value)	Tumor regression (P-value)	Reference
Prostate cancer (PC3)	Athymic nude mice (X)	3×10^6^ (SC)	Upon reaching 50 mm3	10^5^ PFU VSV-hIFNβ	1	NM	No (NM)	(21)
Prostate cancer (RM9)	C57BL/6 (S)	10^4^ (SC)	Upon reaching 50 mm3	10^5^ PFU VSV-mIFNβ	1	NM	No (NM)	(21)
Endometrial adenocarcinoma (AN3 CA)	Athymic nude mice (X)	2×10^6^ (SC)	Upon reaching 0.3-0.5 cm in diameter	10^7^ TCID50 VSV-hIFNβ	1	38 days (0.2628)*	Yes (NM)	(16)
Endometrial adenocarcinoma (HEC-1-A)	Athymic nude mice (X)	2×10^6^ (SC)	Upon reaching 0.3-0.5 cm in diameter	10^7^ TCID50 VSV-hIFNβ	1	60% (0.2028)*	Yes (NM)	(16)
Melanoma (B16-F10)	C57BL/6 (S)	2.5×10^5^ (SC)	Upon reaching ~200 mm3	10^9^ TCID50 VSV-mIFNβ	4	23 days (<0.001)	No (NM)	(28)
Melanoma (B16ova)	C57BL/6 (S)	5×10^5^ (SC)	Upon reaching ~200 mm3	5×10^8^ PFU VSV-mIFNβ	2	NM	Yes (<0.05)	(25)
Colon carcinoma (CT26)	BALB/c (S)	5×10^5^ (SC)	Upon reaching ~200 mm3	10^9^ TCID50 VSV-mIFNβ	4	25 days (<0.05)	Yes (NM)	(28)
Non-small cell lung cancer (LM2)	A/J mice (S)	10^6^ (SC)	NM	1.5×10^10^ TCID50 VSV-mIFNβ	3	30% (<0.001)	Yes (NM)	(15)
Non-small cell lung cancer (H2009)	Nude mice (X)	2.5×10^6^ (SC)	Upon reaching 0.5 cm3	5×10^8^ TCID50 VSV-mIFNβ	3	NM	Yes (NM)	(15)
Non-small cell lung cancer (LM2)	A/J mice (S)	10^6^ (SC)	Upon reaching ~0.5 cm3	1.5×10^9^ TCID50 VSV-mIFNβ	3	25 days (NM)	No (NM)	(27)
Squamous cell carcinoma (FAT-7)	Fischer-344 rats	3×10^6^ (SC)	Upon reaching 0.5 cm in diameter	5×10^8^ PFU VSV-hIFNβ	1	53 days (<0.0001)	Yes (NM)	(17)
Squamous cell carcinoma (FAT-7)	Fischer-344 rats	3×10^6^ (SC)	Upon reaching 0.5 cm in diameter	5×10^7^, 5×10^8^, 5×10^9^ PFU VSV-rIFNβ	1	5×10^7^; 48 days (NM), 5×10^8^; 62 days (0.0008), 5×10^9^; 52 days (NM)	No (NM)	(17)
Squamous cell carcinoma (FAT-7)	Fischer-344 rats	3×10^6^ (SC)	Upon reaching 0.5 cm in diameter	5×10^8^ PFU VSV-rIFNβ	1 and 2	56 days (<0.05)	No (NM)	(17)
Mesothelioma (AB12)	BALB/c (S)	10^6^ (SC)	Upon reaching ~200 mm3	6.6×10^8^ PFU VSV-mIFNβ	3	NM	Yes (<0.01)	(25)
Mesothelioma (AB12)	BALB/c (S)	10^6^ (SC)	Upon reaching ~200 mm3	6.6×10^8^ PFU VSV-hIFNβ	3	NM	NO (0.27)	(25)
Mesothelioma (MSTO)	SCID mice (X)	10^6^ (SC)	Upon reaching ~200 mm3	6.6×10^8^ PFU VSV-mIFNβ	3	NM	NM	(26)
Mesothelioma (REN)	SCID mice (X)	10^6^ (SC)	Upon reaching ~200 mm3	6.6×10^8^ PFU VSV-mIFNβ	3	NM	NM	(26)
Mesothelioma (MSTO)	SCID mice (X)	10^6^ (SC)	Upon reaching ~200 mm3	6.6×10^8^ PFU VSV-hIFNβ	3	NM	NM	(26)
Mesothelioma (REN)	SCID mice (X)	10^6^ (SC)	Upon reaching ~200 mm3	6.6×10^8^ PFU VSV-hIFNβ	3	NM	NM	(26)

X, Xenograft model; S, Syngeneic model; SC, Subcutaneous; NM, Not Mentioned.

### 3.4 Intraperitoneally administered treatment

To establish intraperitoneal tumors, 3.5×10^5^ AB12 cells were injected intraperitoneally in three groups (n=8) of BALB/c models. Upon confirmation of intraperitoneal tumor establishment on day 4, saline or 6.6×10^8^ PFU of each virus (VSV-mIFNβ and VSV-hIFNβ) was injected once a week for 3 weeks. Both VSV-mIFNβ and VSV-hIFNβ significantly prolonged survival compared with saline (p<0.0001 and p<0.01, respectively). A trend toward increased efficacy in VSV-mIFNβ treated mice compared to VSV-hIFNβ treated mice was reported, however, insignificant (p=0.54). Throughout the study, no long-term cures (full recovery) were observed (25).

### 3.5 Intravenously administered treatment

#### 3.5.1 Endometrial adenocarcinoma

Athymic mice with subcutaneously established endometrial adenocarcinoma (AN3 CA) were intravenously administered with one dose of saline, VSV-mIFNβ, or VSV-hIFNβ at 10^6^ TCID_50_ (10 mice per group). All mice in the saline group developed tumors exceeding the limit of 10% of body weight and were therefore euthanized. Of the mice injected with VSV-hIFNβ, one presented with paralysis on day 19 and was euthanized; three others had a tumor weight exceeding the 10% limit of body weight. Both treatments were effective in prolonging survival (pmIFNβ<0.0001, phIFNβ<0.0001, phIFNβ vs. mIFNβ=0.48). The study found that virus levels in the tumor tissue were higher in comparison with brain and blood samples (16).

#### 3.5.2 Lung cancer

LM2 lung cancer was implanted in A/J mice, which were injected with 1×10^6^ murine outgrowth endothelial cells (mBOEC) alone (as a control), 1×10^6^ VSV-IFNβ-infected mBOECs, or 1×10^8^ TCID_50_ VSV-IFNβ on days 20, 22, 24, 41, 43, and 45 in the tail vein and sacrificed on day 48. Although the naked virus did not show a significant difference compared to the control group (p=0.3791), the VSV-IFNβ-infected mBOECs group showed a decreasing trend of lung tumor burden compared to the control (p=0.09). The VSV-N RNA levels from lung homogenates on day 27 were measured and observed to be higher in the VSV-IFNβ-infected mBOEC-treated mice compared to the control (p=0.3) (22).

Fox Chase SCID Beige mice were intravenously implanted with firefly luciferase-expressing A549 cells. fourteen, sixteen, and twenty-nine days post-implantation, mice were systemically injected with saline, 1×10^6^ mBOECs, VSV-IFNβ at 1×10^8^ TCID_50_, or 1×10^6^ VSV-IFNβ-infected mBOECs. VSV-IFNβ-infected mBOECs were more potent at prolonging survival than all other treatments (p<0.001). VSV-IFNβ showed some efficacy compared to controls; however, increased toxicity were reported, resulting in early death. The naked VSV-IFNβ-treated mice lost weight and were not as active as they normally were; yet, there was no limb paralysis, decreasing the likelihood of the effects being induced by neurotoxicity (22).

#### 3.5.3 Squamous cell carcinoma

FAT-7 tumors were established in immunocompetent Fisher 344 rats until they became palpable (approximately 0.5 cm in diameter). One VSV-rIFNβ dose of 5×10^8^ PFU was intravenously administered in 10 tumor-bearing rats. Compared with saline controls (n=10), VSV-rIFNβ significantly delayed tumor growth (p<0.0001 on day 43 post-treatment) and prolonged survival (p=0.0084). Moreover, another group of FAT-7 tumor-bearing rats were treated with a single dose of 5×10^8^ PFU VSV-hIFNβ or saline (10 per group) at about 21 days post-tumor implantation (approximately 0.5 cm in diameter). On day 38, there was a significant improvement in both survivalsurvivals (p<0.0001) and tumor growth-delaying effects (p=0.0004) compared to the control (17).

#### 3.5.4 Plasmacytoma

BALB/c mice with subcutaneously implanted MPC-11 plasmacytomas were intravenously treated with one dose of 2×10^6^, 2×10^7^, or 2×10^8^ TCID_50_ VSV expressing IFNβ and sodium iodine symporter (VSV-IFNβ-NIS), which is a reporting gene used to facilitate the imaging of viral spread.-mIFNβ-NIS. Dose-dependent tumor regression or growth inhibition rapidly became evident but did not differ from that in the controls. Despite the strong response to the treatment, adverse effects began to arise and soon became lethal thereafter. By day 3, the VSV-treated groups had lost 15–30% of their body weight possibly as a consequence of insufficient water intake and anorexia. Other adverse effects included inactivity, shivering, hypothermia, scruffy coat development, and early death. Most mice did not survive beyond day 9 post-injection. At no point during the study were neurotoxicity and hindlimb paralysis or any of their signs observed. Nonetheless, the survival curves of the treatment groups did not differ from those of the saline controls (29).

Postmortem necropsy of the liver revealed high occurrence rates of intravascular coagulopathy. The mice treated with VSV-mIFNβ and VSV-mIFNβ-NIS (n=4) showed white blood cell and platelet counts of 0.67 ± 0.14×10^6^/μL and 104.2 ± 18.1×10^6^/μL compared with 12.5 ± 2.3×10^6^/μL and 561.3 ± 23.2×10^6^/μL in the saline models (n=3), respectively. The tumor sizes were compared to evaluate further the factors inducing adverse events. The mice with small tumors (15.9 ± 3.7 mm^3^, n=10) or larger tumors (55.6 ± 15 mm^3^, n=10) were intravenously treated with 1×10^7^ TCID^50^ VSV-mIFNβ-NIS. The mice with larger tumors exhibited more notable lymphopenia and thrombocytopenia than those with smaller tumors (29).

C57Bl/KaLwRji mice were implanted with 5TGM1 myeloma tumors subcutaneously. Fourteen days post-implantation, mice were injected with a single dose of 1×10^8^ TCID_50_ VSV-mIFNβ, VSVhIFNβ, or saline. All tumors treated with VSV-mIFNβ showed tumor regression, whereas 80% of mice treated with VSV-hIFNβ demonstrated regression. Both groups showed significantly longer survival intervals (pVSV-mIFNβ=0.0018, pVSV-hIFNβ=0.04) than the saline group. No associated toxicity was observed at this dose level. Upon histopathological analysis, no viral particles were detectable in the brain, suggesting high safety profile at this dose of intravenous administration (8).

Orthotopic 5TGM1 myeloma was intravenously established in C57Bl/KaLwRji mice, and a single dose of 5×10^7^ TCID_50_ VSV-mIFNβ or VSV-hIFNβ or 100 µL saline was intravenously injected in the tail. The treatments significantly prolonged the survival of the myeloma-bearing mice (pVSV-mIFNβ=0.0008, pVSV-hIFNβ=0.017). Furthermore, VSV-mIFNβ significantly prolonged the survival compared with VSV-hIFNβ (p=0.021), with one mouse becoming completely disease-free (8).

Further, in another experiment, 5TGM1 myeloma cells were subcutaneously implanted in C57BL/KALwRij mice. PBS (n=7) or 1×10^8^ TCID_50_ VSV-mIFNβ-NIS (n=10) was intravenously injected. The treatment group survived significantly longer than the control group (p=0.0084) (18).

Moreover, 5TGM1 tumor cells were intravenously injected into C57BL/KaLwRij mice, and 28 days later, either 1×10^8^ TCID_50_ VSV-mIFNβ-NIS (n=5) or PBS (n=5) was intravenously injected as a treatment. As opposed to the results of the subcutaneous tumor-implanted mice, there was no significant difference in survival between the treatment and control groups (p=0.332). The majority of the mice in the treatment group (56%) were euthanized owing to isolating or lethargic behavior. These mice exhibited labored breathing, isolated themselves to a corner of their cage, did not eat or drink, and therefore lost weight rapidly. The death of the mice with systemic myeloma was hypothesized to be related to VSV-induced neurotoxicity instead of tumor burden (18).

C57BL/KaLwRij female mice were subcutaneously implanted with syngeneic 5TGM1 plasmacytomas and were treated with 1×10^5^, 1×10^6^, 1×10^7^, or 1×10^8^ TCID_50_ of VSV-mIFNβ-NIS. As expected, the 1×10^8^ TCID_50_-treated mice experienced the longest remission before relapse. Survival was significantly prolonged in all treatment groups (p1e5 = 0.0491, p1e6 = 0.0178, p1e7<0.0001, p1e8<0.0001). Groups treated with 1×10^7^ and 1×10^8^ TCID_50_ exhibited significantly prolonged survival compared with the lower dose groups (p=0.0008). The median survival of the control and four treatment groups in order of increasing dose was 19, 20, 26, and 30.5 days, respectively. All experimental groups exhibited transient weight loss, which peaked on day 1 in the rodents injected with 1×10^8^ TCID_50_ and recovered by day 5. Of the 50 mice, 42 were euthanized for tumor burden, 4 for 50% tumor ulceration, and 2 for lethargy, and 2 died (23).

KAS6/1 myeloma xenografts were subcutaneously implanted in CB17 ICR SCID mice and were intravenously treated with 1×10^5^, 1×10^6^, 1×10^7^, or 1×10^8^ TCID_50_ VSV-mIFNβ-NIS. VSV-mIFNβ-NIS showed an effective tumor control at all dose levels (p<0.0001) and significantly increased the survival rate in comparison to control (p1e5 = 0.0047, p1e6 = 0.0047, p1e7<0.0001, p1e8 = 0.0002). Six mice, one of which was from the control group, were euthanized after 84 to 118 days post-treatment owing to hindlimb paralysis. This might have been caused by the spread of the myeloma into the bone marrow of the mice (23).

#### 3.5.5 Acute myeloid leukemia

C1498 cells were subcutaneously injected into C57BL/6J mice. Fourteen days post tumor implantation, a single dose of 1×10^6^, 1×10^7^, or 1×10^8^ TCID_50_ VSV-mIFNβ-NIS, or saline was administered intravenously. Tumor weights in treatment groups were significantly lower than control (1×10^6^; p=0.0094, 1×10^7^ and 1×10^8^; p<0.0001). Higher doses showed lower tumor weights compared to lower doses (1×10^6^ vs. 1×10^7^; p=0.0432) (24).

In another set of C57BL/6J mice, C1498.GFP cells were injected systemically. To facilitate the detection of cells, GFP protein was transduced into the C1498 cells. Twelve days post-implantation, upon identification of C1498.GFP cells in the blood, liver, spleen, and bone marrow, one dose of 1×10^8^ TCID_50_ VSV-mIFNβ-NIS or saline was injected intravenously. significantly prolonged survival was reported in the treatment group (p=0.0043; compared to control) (24). The studies that utilized the intravenous treatment of VSV-IFNβ are summarized in **Table 4**.

**Table 4:** Summary of the studies that utilized intravenous treatment.

Tumour type (cell line)	Animal model (X/S)	Number of seeded tumor cells (route)	Time of treatment in relation to tumor implantation	Treatment concentration	Number of doses	Survival benefits (P-value)	Tumor regression (P-value)	Reference
Endometrial adenocarcinoma (AN3 CA)	Athymic nude mice (X)	2×10^6^ (SC)	Upon reaching 0.3-0.5 cm in diameter	10^6^ TCID50 VSV-mIFNβ	1	70% (<0.0001)	Yes (NM)	(16)
Endometrial adenocarcinoma (AN3 CA)	Athymic nude mice (X)	2×10^6^ (SC)	Upon reaching 0.3-0.5 cm in diameter	10^6^ TCID50 VSV-hIFNβ	1	60% (<0.0001)	Yes (NM)	(16)
Lung cancer (LM2)	A/J mice (S)	2×10^5^ (IV)	20, 22, 24, 41, 43, and 45 days post-implantation	10^8^ TCID50 VSV-mIFNβ	6	NM	NM	(22)
Lung cancer (LM2)	A/J mice (S)	2×10^5^ (IV)	20, 22, 24, 41, 43, and 45 days post-implantation	10^6^ VSV-mIFNβ-infected mBOECs	6	NM	NM	(22)
Lung cancer (Luc-A549))	Fox Chase SCID Beige (X)	10^6^ (IV)	14, 16, and 29 days post-implantation	10^8^ TCID50 VSV-mIFNβ	3	42 days (NM)	No (NM)	(22)
Lung cancer (Luc-A549))	Fox Chase SCID Beige (X)	10^6^ (IV)	14, 16, and 29 days post-implantation	10^6^ VSV-mIFNβ-infected mBOECs	3	54 days (<0.001)	No (NM)	(22)
Squamous cell carcinoma (FAT-7)	Fischer-344 rats (S)	3×10^6^ (SC)	Upon reaching 0.5 cm in diameter	5×10^8^ PFU VSV-rIFNβ	1	53 days (0.0084)	No (NM)	(17)
Squamous cell carcinoma (FAT-7)	Fischer-344 rats (S)	3×10^6^ (SC)	Upon reaching 0.5 cm in diameter	5×10^8^ PFU VSV-hIFNβ	1	53 days (<0.0001)	Yes (NM)	(17)
Myeloma (5TGM1)	C57BL/KaLwRij (S)	5×10^6^ (SC)	14 days post-implantation	10^8^ TCID50 VSV-mIFNβ-NIS	1	55% (0.0084)	NM	(18)
Myeloma (5TGM1)	C57BL/KaLwRij (S)	10^7^ (IV)	28 days post-implantation	10^9^ TCID50 VSV-mIFNβ-NIS	1	9 days (0.332)	NM	(18)
Myeloma (KAS6/1)	CB17 ICR SCID mice (X)	10^7^ (SC)	Upon reaching 5 mm in length or width	10^5^, 10^6^, 10^7^, or 10^8^ TCID50 VSV-mIFNβ-NIS	NM	10^5^; 60% (0.0047), 10^6^; 50% (0.0047), 10^7^; 70% (<0.0001), 10^8^; 60% (0.0002)	No (NM)	(23)
Myeloma (5TGM1)	C57BL/KaLwRij (S)	5×10^6^ (SC)	Upon reaching 5 mm in length or width	10^5^, 10^6^, 10^7^, or 10^8^ TCID50 VSV-mIFNβ-NIS	1	10^5^; 19 days (0.0491), 10^6^; 20 days (0.0176), 10^7^; 26 days (<0.0001), 10^8^; 30.5 days (<0.0001)	Yes (NM)	(23)
Myeloma (MPC-11)	Balb/c (S)	5×10^6^ (SC)	NM	2×10^6^, 2×10^7^,or 2×10^8^ TCID50 VSV-mIFNβ-NIS	1	Insignificant	Yes (NM)	(29)
Myeloma (5TGM1)	C57BL/KaLwRij (S)	5×10^6^ (SC)	14 days post-implantation	10^8^ TCID50 VSV-mIFNβ	1	Prolonged (0.0018)	Yes (NM)	(8)
Myeloma (5TGM1)	C57BL/KaLwRij (S)	5×10^6^ (SC)	14 days post-implantation	10^8^ TCID50 VSV-hIFNβ	1	Prolonged (0.04)	Yes (NM)	(8)
Myeloma (5TGM1)	C57BL/KaLwRij (S)	5×10^6^ (IV)	21 days post-implantation	5×10^7^ TCID50 VSV-mIFNβ	1	45 days (0.0008)	Yes (NM)	(8)
Myeloma (5TGM1)	C57BL/KaLwRij (S)	5×10^6^ (IV)	21 days post-implantation	5×10^7^ TCID50 VSV-hIFNβ	1	35 days (0.017)	No (NM)	(8)
Acute myeloid leukemia (C1498)	C57BL/6J (S)	2×10^6^ (SC)	14 days post-implantation	10^6^, 10^7^, or 10^8^ TCID50 VSV-mIFNβ-NIS	1	NM	NM	(24)
Acute myeloid leukemia (C1498.GFP)	C57BL/6J (S)	2×10^6^ (IV)	12 days post-implantation	10^8^ TCID50 VSV-mIFNβ-NIS	1	11 days (0.0043)	NM	(24)

X, Xenograft model; S, Syngeneic model; SC, Subcutaneous; IV, Intravenously; NM, Not Mentioned.

The above results showed the potential efficacy of using VSV-IFNβ *via* different administration routes as a virotherapeutic agent, albeit intravenous route impose some difficulties such as neutralization by antiviral antibodies (32). The efficacy of both administration routes are comparable; 20 intratumoral experiments out of 23 (86.956%) displayed efficacious outcomes compared to 15 out of 17 (88.235%) for the intravenously administered study groups. Intraperitoneal administrations were 2 out of 2 positive results. Furthermore, various VSV-IFNβ constructs were used in these experiments, origins of the constructs and its outcomes are illustrated in section 3.6.

### 3.6 Virus constructs

Among the 14 included articles, numerous viral constructs have been utilized, seven of which used the same construct previously described (9). Three articles used three different virus constructs, and four other articles did not mention the origin of their viral construct. Detailed comparison between the constructs and their outcomes has been embraced in **Table 5**.

**Table 5:** Summary of the VSV constructs of the reviewed studies.

First Author, Year of Publication	Reference	Type of VSV	VSV Construct	Route of Administration	Outcome
Udayakumar et al., 2020	(21)	VSV-hIFNβ	NM	IT	–
		VSV-mIFNβ	NM	IT	+
Liu et al., 2014	(16)	VSV-hIFNβ	Obuchi, 2013	IT	+
			Obuchi, 2013	IT	+
			Obuchi, 2013	IV	+
		VSV-mIFNβ	Obuchi, 2013	IV	+
Patel et al., 2020	(22)	VSV-mIFNβ	NM*	IV	–
			NM*	IV	+
Zhang et al., 2016	(23)	VSV-mIFNβ-NIS	Werts, 1995	IV	+
			Wertz, 1995	IV	+
Shen et al., 2016	(24)	VSV-mIFNβ-NIS	Naik, 2012	IV	+
			Naik, 2012	IV	+
Willmon et al., 2009	(25)	VSV-hIFNβ	Obuchi, 2013	IT	–
			Obuchi, 2013	IP	+
			Obuchi, 2013	IT	+
		VSV-mIFNβ	Obuchi, 2013	IT	+
			Obuchi, 2013	IT	+
			Obuchi, 2013	IT	+
			Obuchi, 2013	IT	+
			Obuchi, 2013	IT	+
			Obuchi, 2013	IP	+
Patel et al, 2015	(15)	VSV-mIFNβ	Obuchi, 2013	IT	+
			Obuchi, 2013	IT	+
Kurisetty et al, 2014	(17)	VSV-hIFNβ	Obuchi, 2013	IV	+
			Obuchi, 2013	IT	+
		VSV-rIFNβ	Obuchi, 2013	IT	+
			Obuchi, 2013	IT	+
			Obuchi, 2013	IV	+
Saloura et al, 2010	(26)	VSV-hIFNβ	Obuchi, 2013	IT	–
			Obuchi, 2013	IT	+
		VSV-mIFNβ	Obuchi, 2013	IT	+
			Obuchi, 2013	IT	+
Patel et al, 2019	(27)	VSV-mIFNβ	NM*	IT	+
Durham et al, 2017	(28)	VSV-mIFNβ	NM	IT	+
			NM	IT	+
Naik et al, 2012	(8)	VSV-hIFNβ	Obuchi, 2013	IV	+
			Obuchi, 2013	IV	+
		VSV-mIFNβ	Obuchi, 2013	IV	+
			Obuchi, 2013	IV	+
Zhang et al, 2016	(29)	VSV-mIFNβ-NIS	Obuchi, 2013	IV	+
Yarde et al, 2013	(18)	VSV-mIFNβ-NIS	Russel, 2010	IV	+
			Russel, 2010	IV	–

NM, Not Mentioned; IV, Intravenous; IP, Intraperitoneal; IT, Intratumoral; +, the study reported a positive outcome; -, the study reported a negative outcome; *: both studies denoted catalog numbers (hIFNβ, cat. no. OV2010; mIFNβ, cat. no. OV2014), yet no source was mentioned.

## 4 Discussion

This narrative review focused on the efficacy of VSV-IFNβ in multiple established tumors by systematically searching for *in vivo* preclinical studies that utilized VSV-IFNβ and comparing its efficacy to that of non-treatment controls. The Medline and Embase databases were searched in detail for relevant published articles. A total of 14 articles met the eligibility criteria and were finally included. The results of 4238 experiments were classified and presented on the basis of the route of VSV-IFNβ administration: intratumoral, intraperitoneallocoregional, and intravenous. This review highlights the potential safety and efficacy of VSV-IFNβ arising from the defective IFN pathway that substantially impacts the ability of tumor defense mechanisms to establish an effective IFN response (9).

Oncolytic viruses have attracted marked attention owing to their ability to induce tumor cell death while leaving healthy cells unharmed. However, there are a number of obstacles to overcome to translate their potential efficacy into clinical practice. These challenges include the optimization of systemic delivery of oncolytic viruses, tumor virus dispersion, and anti-tumor immune cross-priming. The key factor in overcoming these challenges is optimizing an animal cancer model with a tumor microenvironment as close as possible to human cancers. Xenograft cell line implantation may provide a more efficient model to tackle the aforementioned impediments. Nevertheless, a key consequence of cancer cell engraftment into immunocompetent models is rapid immune rejection (33). However, immunosuppressed models offer a possible xenograft model. This may affect the ability of oncolytic viruses to induce anti-cancer immune cross-priming. Additionally, when cultured *in vitro* for a long period, cell lines may acquire additional mutations, deviating from their similarity to human tumor morphology and heterogeneity (34).

Although IFN-β has a favorable safety profile, neurotoxicity remains a concern, particularly in immunodeficient mice (9, 16, 25). Euthanasia due to neurotoxicity has been reported in SCID mice with xenograft mesothelioma treated with VSV-hIFN-β, while mice treated with mIFN-β did not experience neurotoxicity (25). However, this may be due to the biologically inactive human IFN-β in mice (25). Another study also reported neurotoxicity following VSV-hIFN-β administration in xenograft models, which supports the added safety of IFN-β expression (16). In general, VSV-mIFN-β was well tolerated in all studies except one (9, 16, 25). Neurotoxicity was reported in systemically established myeloma models, but not in subcutaneously established myeloma models (18). Yarde et al. suggest that the observed neurotoxicity was caused by the neurovirulence of VSV-mIFN-β (18). In contrast, Zhang et al. found that neurotoxicity was not observed in tumor-bearing or non-tumor-bearing mice (immunocompetent or SCID mice) following treatment with VSV-IFNβ-NIS (23). These findings suggest that VSV-mIFN-β has the potential as an oncolytic virotherapeutic agent, but further research is needed.

Intravenous virus delivery allows the targeting of visceral tumors. However, antiviral antibodies and complement system components constitute a major barrier by reducing the viral load (32). Solutions to avoid this include immunosuppression or viral modification, shielding the virus from neutralizing antibodies (35–38). Since virally induced immune responses constitute a part of virotherapeutic efficacy, immune suppression might reduce the overall efficacy of the oncolytic virus. This allows VSV-IFNβ to elicit a strong immune response in infected cells, including the production of IFNβ and the activation of immune cells such as natural killer (NK) cells and T cells (9, 28, 39). However, the immune system can also mount a counter-immune response to VSV-IFNβ through several mechanisms. One mechanism is the production of neutralizing antibodies, which can bind to and inhibit the activity of VSV (40). This can occur as a result of prior exposure to VSV or to other viruses that have similar epitopes, or it can be induced by vaccination with a VSV-based vaccine. Another mechanism of counter-immune response is the upregulation of PDL-1, which can suppress the immune response to VSV-IFNβ and limit its effectiveness (15). However, these characteristics could potentiate the use of checkpoint inhibitors as a potential combination therapy.

Meanwhile, cellular carriers may provide a solution. Several types of cellular carriers have been utilized, including T cells, mesenchymal stem cells, cancer cells, and blood outgrowth endothelial cells (BOECs) (22, 40–44). Cellular carriers must be optimized to be efficiently infected by the oncolytic virus and carry the virus selectively to the tumor microenvironment, shielding it from immunological recognition. BOECs carrying VSV-IFNβ have been effectively utilized in a non-small-cell lung cancer model (22). Accordingly, a promising opportunity to mitigate the above-mentioned issue may be offered by cellular carriers.

Combination therapy VSV-IFNβ with checkpoint inhibitors, radiotherapy (RT), or JAK/STAT inhibition offers a higher treatment potential profile than does VSV-IFNβ monotherapy (21, 24, 27). Through combination with RT, an enhancement of VSV-induced oncolysis was reported (21). Even in resistant prostate cancer cell lines, a combination of VSV-IFNβ and RT has been shown to upregulate pro-apoptotic genes and suppress antiviral/apoptotic genes. VSV replication has been enhanced upon the addition of RT to VSV-IFNβ. *In vivo*, resistant prostate tumor xenograft models showed higher susceptibility to VSV-IFNβ+RT than to VSV-IFNβ alone. Moreover, syngeneic prostate cancer models showed a complete response to VSV-IFNβ+RT and were rechallenged; 100% tumor rejection was observed. However, upon administration of anti-CD8 antibodies, tumor growth was reported, suggesting that anti-tumor immunity is induced through CD8+ lymphocytes (21). Further, inhibition of JAK/STAT using ruxolitinib in combination with VSV-IFNβ improved the survival of non-small-cell lung cancer models when compared with inhibition using VSV-IFNβ or ruxolitinib alone (27). Similarly, in acute myeloid leukemia models, the combination of VSV-IFNβ-NIS with an anti-PD-L1 antibody enhanced tumor regression compared with VSV-IFNβ-NIS alone (24).

A possible impeding factor of the anti-tumor immune response is the Warburg effect. As aggressively proliferating tumor cells consume glucose within the tumor microenvironment through glycolysis, immune effector cells, including T cells, are rendered defective owing to glucose depletion (45, 46). The accumulation of lactate in the tumor microenvironment adversely affects the functionality of T and NK cells, contributing to the immunosuppression of the tumor microenvironment (47, 48). Thus, inhibiting glycolysis has been observed to enhance anti-tumor immunity (49, 50). A possible approach to improve the efficacy of virotherapy is the combination with immune modulators, such as dichloroacetate (DCA). By inhibiting lactate generation, DCA is reported to shift the metabolism of tumor cells to oxidative phosphorylation, restoring glucose reservoirs for immune cells and thus enhancing the immune function in the immune microenvironment (51).

The specific mechanism of action of VSV-IFNβ, along with its broad antiviral activity, low toxicity, and ability to replicate in a wide range of cell types, makes it a promising candidate for cancer therapy (5, 52). VSV-IFNβ has demonstrated safety and effectiveness in preclinical models and in human subjects, making it a potential candidate for further clinical development (53–55). It may also have potential applications in the treatment of a specific type of cancer or in combination with other therapies. In comparison to other modified oncolytic viruses under study or approved for use, VSV-IFNβ stands out for its unique mechanism of action and promising results in preclinical and clinical studies. For example, some other modified oncolytic viruses have been developed to target specific types of cancer or to express immunostimulatory molecules such as cytokines or tumor antigens. While these approaches may be effective in certain contexts, VSV-IFNβ’s ability to express IFNβ and elicit a broad immune response may make it more versatile and effective in a wider range of cancer types.

Although this review was performed by exhaustively searching two relevant databases for eligible studies, non-English-language articles were excluded owing to technical difficulties in acquiring accurate data. This review was conducted to provide a collective reference for researchers who are seeking to investigate further the efficacy and safety of VSV-IFNβ and the potential to translate this treatment into clinical settings.

## 5 Conclusion

While IFN-β has a generally favorable safety profile, neurotoxicity has been reported in some studies of VSV-IFNβ treatment. This neurotoxicity may be more pronounced in immunodeficient mice, and may be due to the biologically inactive human IFN-β in mice. However, other studies have found that VSV-mIFN-β was well tolerated in all tested models, with neurotoxicity only being observed in certain contexts, such as in systemically established myeloma models. Overall, further research is needed to fully understand the potential side effects of VSV-IFNβ treatment and to optimize its use as an oncolytic virotherapeutic agent.

## Data availability statement

The original contributions presented in the study are included in the article/supplementary material. Further inquiries can be directed to the corresponding authors.

## Author contributions

AA and ABM contributed to the conception and design of the study. AMM, HA, OA, and YS performed title, abstract, and full-text screening and data extraction of included studies. Conflicts were resolved by FA. YS and OA performed SYRCLE’s RoB assessment tool; conflicts were resolved by AA. AMM, FA, and OA wrote the first draft of the manuscript and the sections of the manuscript. All authors contributed to the manuscript revision, read, and approved the submitted version.
